# A Non-canonical Voltage-Sensing Mechanism Controls Gating in K2P K^+^ Channels

**DOI:** 10.1016/j.cell.2016.02.002

**Published:** 2016-02-25

**Authors:** Marcus Schewe, Ehsan Nematian-Ardestani, Han Sun, Marianne Musinszki, Sönke Cordeiro, Giovanna Bucci, Bert L. de Groot, Stephen J. Tucker, Markus Rapedius, Thomas Baukrowitz

**Affiliations:** 1 Institute of Physiology, Christian-Albrechts University, 24118 Kiel, Germany; 2 Computational Biomolecular Dynamics Group, Max Planck Institute for Biophysical Chemistry, 37077 Göttingen, Germany; 3 Leibniz-Institut für Molekulare Pharmakologie, 13125 Berlin, Germany; 4 Clarendon Laboratory, Department of Physics, University of Oxford, Oxford OX1 3PU, UK; 5 OXION Initiative, University of Oxford, Oxford OX1 3PU, UK; 6 Nanion Technologies GmbH, 80636 Munich, Germany

## Abstract

Two-pore domain (K2P) K^+^ channels are major regulators of excitability that endow cells with an outwardly rectifying background “leak” conductance. In some K2P channels, strong voltage-dependent activation has been observed, but the mechanism remains unresolved because they lack a canonical voltage-sensing domain. Here, we show voltage-dependent gating is common to most K2P channels and that this voltage sensitivity originates from the movement of three to four ions into the high electric field of an inactive selectivity filter. Overall, this ion-flux gating mechanism generates a one-way “check valve” within the filter because outward movement of K^+^ induces filter opening, whereas inward movement promotes inactivation. Furthermore, many physiological stimuli switch off this flux gating mode to convert K2P channels into a leak conductance. These findings provide insight into the functional plasticity of a K^+^-selective filter and also refine our understanding of K2P channels and the mechanisms by which ion channels can sense voltage.

## Highlights

• Most K2P channels exhibit strong voltage gating and are not simple leak channels
• Voltage sensing involves movement of K^+^ into the electric field of an inactive filter
• MD simulation of permeation reveals insight into the filter gating mechanism
• Many physiological stimuli modulate this voltage-gating behavior

## Graphical Abstract

**Figure:**
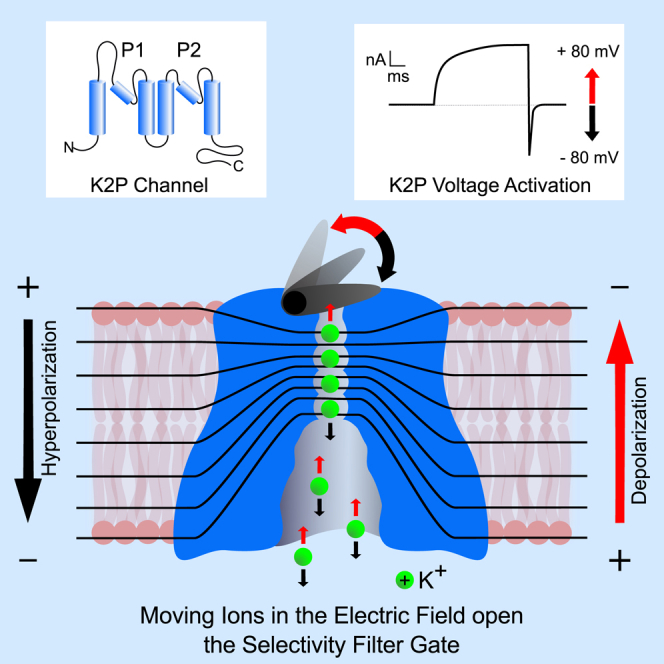

## Teaser

K2P channels, which do not possess a canonical voltage-sensing domain, can be voltage gated by an ion check valve mechanism.

## Introduction

In the animal kingdom three main families of K^+^ channels define K^+^-dependent cellular excitability, namely, the voltage-gated (Kv), inwardly rectifying (Kir), and the background or leak two-pore domain (K2P) K^+^ channels. Kv channels contribute to membrane repolarization; they are gated open by membrane depolarization, which is sensed by a positively charged “S4-helix” within a canonical voltage-sensing domain (VSD). Kir channels stabilize the resting membrane potential (RMP) and exhibit a strong voltage-dependent behavior because they are blocked by polyamines like spermine upon depolarization. By contrast, K2P channels have traditionally been viewed as voltage-independent “background” K^+^ channels where their strong outward rectification is thought to arise from the asymmetric K^+^ gradient across the membrane as predicted by the Goldman-Hodgkin-Katz (GHK) equation (Goldstein et al., 2001, Lesage and Lazdunski, 2000). This outward rectification has important implications for the role of K2P channels in the central and peripheral nervous system because it not only stabilizes the RMP but also contributes to repolarization and even enables action potential generation in the absence of classical Kv channels (MacKenzie et al., 2015). Such properties are greatly enhanced in some K2P channels (in particular TREK-1 and TASK-3) because of additional time- and voltage-dependent activation (Bockenhauer et al., 2001, Brickley et al., 2007). However, K2P channels lack a VSD, and the mechanisms that underlie this voltage-gating behavior remain unclear.

There are 15 members of the human K2P family that can be divided into six subfamilies based on their structural and functional properties (Goldstein et al., 2005, Lesage and Lazdunski, 2000), namely, the TWIK, TREK, TASK, THIK, TALK, and TRESK subfamilies (Enyedi and Czirják, 2010). Recent crystal structures demonstrate that, unlike other classical tetrameric K^+^ channels, K2P channels assemble as dimers with a pseudotetrameric pore (Brohawn et al., 2012, Brohawn et al., 2013, Dong et al., 2015, Miller and Long, 2012). Extensive studies over the last two decades have revealed that these channels represent major regulators of cellular excitability and are involved in a wide range of physiological functions, including vasodilation, neuroprotection, anesthesia, sleep, chemo- and nutrient sensing, aldosterone and insulin secretion, nociception, and pressure and temperature sensing (Enyedi and Czirják, 2010, Honoré, 2007).

Requisite for such functional diversity is their regulation by a wide variety of stimuli, including PUFAs (e.g., arachidonic acid, AA), lysophospholipids, DAG, phosphoinositides (e.g., PIP_2_), phosphorylation, volatile anesthetics, pH changes, temperature, and mechanical forces (Enyedi and Czirják, 2010, Honoré, 2007). For some of these stimuli, the molecular determinants have been partially identified and involve a gating machinery located within the selectivity filter (SF) (Bagriantsev et al., 2011, Lolicato et al., 2014, Piechotta et al., 2011, Zilberberg et al., 2001). But exactly what happens within the filter during K2P channel gating remains unknown.

Here, we show that voltage-dependent gating is a property common to nearly all K2P channels. It originates from the unique functional plasticity of the selectivity filter and represents a significant component of their outward rectification. These results expand our understanding of the mechanisms that underlie voltage sensing in ion channels and the functional role that K2P channels may play in cellular excitability.

## Results

### Voltage Activation Is a Common Feature in Almost all K2P Channels

**Figure 1:**
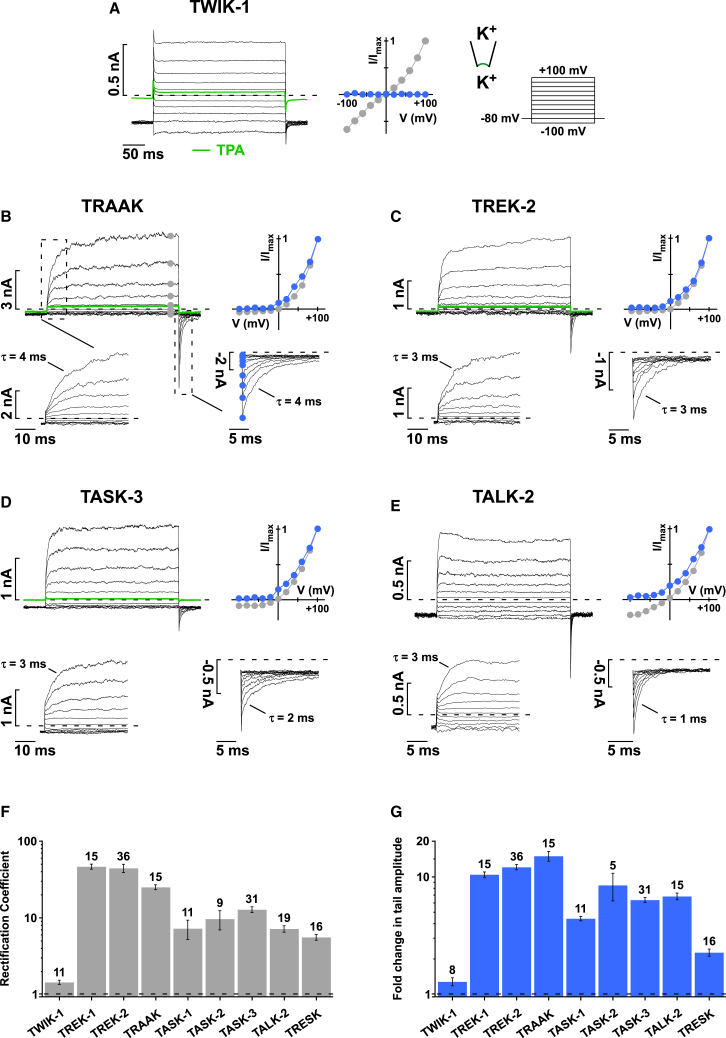
Voltage Gating Is Common within the K2P Superfamily (A–E) Current responses to a 300-ms voltage step family from −100 to +100 mV from a holding potential of −80 mV recorded from excised inside-out membrane patches in symmetrical K^+^ (120 mM K^+^_int._/120 mM K^+^_ext._) expressing the indicated K2P channel with (A) TWIK-1, (B) TRAAK, (C) TREK-2, (D) TASK-3, and (E) TALK-2; the current at +100 mV after K2P channel block by 1 mM TPA is shown in green (Piechotta et al., 2011). The I-V plots indicate currents at the end of the depolarizing steps (gray circles) and the corresponding inward current (tail) amplitudes upon repolarization to −80 mV (blue circles); the insets show the time course of voltage activation and inactivation with higher time resolution; time constants (τ) are obtained with exponential fits. (F and G) Rectification coefficients (currents at +100mV/–100mV) (F) and fold change in tail current amplitudes (G) subsequent to a depolarizing pulse to +100 mV for the indicated K2P channels; data are represented as mean ± SEM. See also Figures S1 and S2.

**Figure S1:**
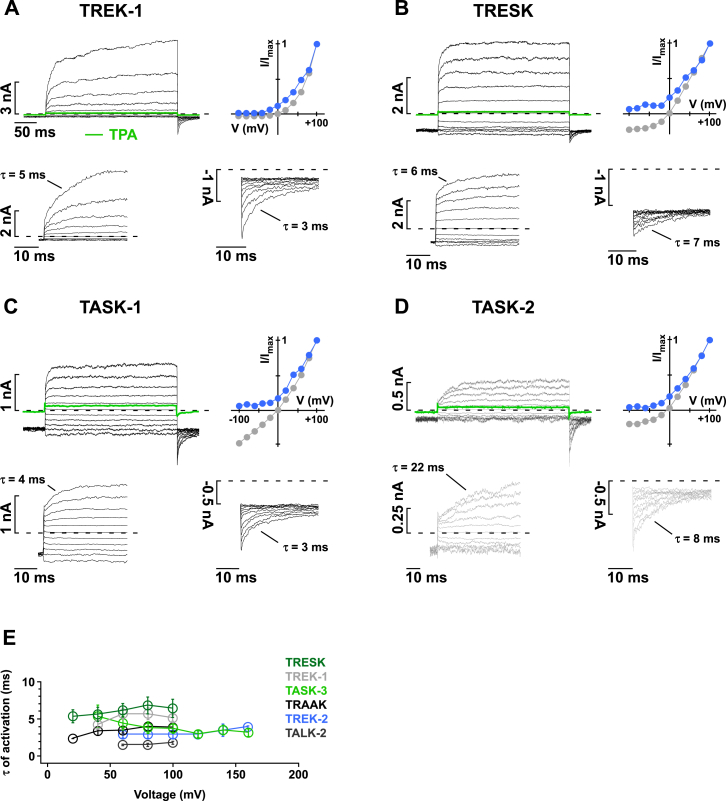
Voltage Gating in TREK-1, TRESK, TASK-1, and TASK-2 Channels and K2P Channel Gating Kinetics, Related to Figure 1 (A–D) Current responses to a family of 300 ms voltage steps from −100 to +100 mV from and to a holding potential of −80 mV recorded from inside-out patches excised from *Xenopus* oocytes in symmetrical K^+^ (120 mM [K^+^]_int._/120 mM [K^+^]_ext._) expressing TREK-1, (B) TRESK, (C) TASK-1 and (D) TASK-2 channels. I-V plots indicate the currents at the end of the depolarizing steps (gray circles) and inward current (tail) amplitudes upon repolarization to −80 mV (blue circles); the insets show the time course of voltage activation and deactivation with higher time resolution as indicated by the scale bars; time constants (τ) revealed by exponential fit functions. (E) Summary of time constants for voltage activation obtained for different channels and voltages. Data are represented as mean ± SEM.

To obtain a comprehensive picture of this voltage-dependent gating, we measured currents in response to 300-ms voltage steps (from −100 to +100 mV) for various members of the K2P channel family in excised giant membrane patches under symmetrical K^+^ conditions (Figures 1 and S1). TWIK-1 channels display linear current-voltage characteristics (I-V curves) and no sign of time- or voltage-dependent activation (Figure 1A). In marked contrast, all other tested K2P channels (TRAAK, TREK-2, TREK-1, TRESK, TALK-2, TASK-1, TASK-2, and TASK-3) showed prominent outward rectification as a result of a time- and voltage-dependent activation process (Figures 1B–1E and S1A–S1D). In TRAAK, for instance, only very small currents were observed at negative potentials, but large outward currents developed for voltage steps positive to the reversal potential (E_rev._). This voltage activation proceeded with a time constant (τ) of 4 ± 1 ms that was strikingly voltage independent (i.e., similar for potentials between +20 to +100 mV; Figures 1B and S1E).

Most remarkable, however, was the appearance of large inward (i.e., tail) currents upon repolarization to −80 mV that indicate a large rise in open probability (*P*_*O*_). These tail currents increased in parallel with the amplitudes of the outward currents and decayed with a time constant of 4 ± 2 ms (Figure 1B). Similar tail currents were observed for TREK-1 and TREK-2 (Figures 1C and S1A), TALK-2 (Figure 1E), TASK-2 (Figure S1D), and TASK-3 channels (Figure 1D). The degree of outward rectification was less prominent in TRESK and TASK-1 as these channels conducted larger inward currents at negative potentials. Nevertheless, TRESK and TASK-1 also showed significant time-dependent voltage activation with corresponding tail currents seen upon repolarization (Figures S1B and S1C). The degree of outward rectification and fold change in tail current amplitudes upon depolarization are summarized for all tested channels in Figures 1F and 1G. This establishes that time- and voltage-dependent activation is a prominent feature within the K2P superfamily. Interestingly, the only K2P channel that behaved like a “classical” leak channel with a linear I-V curve was TWIK-1.

**Figure S2:**
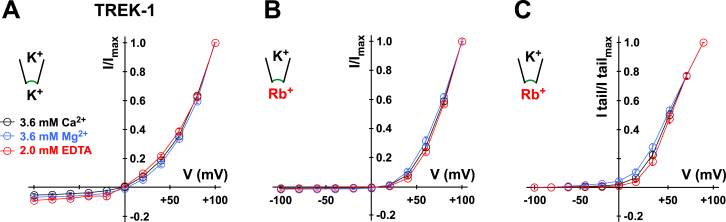
Extracellular Divalent Cations Have No Marked Effect on Voltage Gating, Related to Figure 1 (A) I-V plots of TREK-1 currents in symmetrical K^+^ (120 mM [K^+^]_int._/120 mM [K^+^]_ext._) obtained from voltage families in the presence of extracellular 3.6 mM Ca^2+^ or 3.6 mM Mg^2+^ and in the absence of divalent cations with 2 mM EDTA added. (B) Protocol as in (A) but with Rb^+^ at the intracellular side (120 mM [Rb^+^]_int._/120 mM [K^+^]_ext._). (C) The I-V plots show the normalized inward current (tail) amplitudes subsequent to 300 ms pre-pulse voltage steps to the indicated voltage with Rb^+^ as the intracellular cation (120 mM [Rb^+^]_int._/120 mM [K^+^]_ext._) in the presence of extracellular 3.6 mM Ca^2+^ or 3.6 mM Mg^2+^ and in the absence of divalent cations with 2 mM EDTA added as indicated. Data are represented as mean ± SEM.

This next raises the question of what mechanisms underlie this voltage-dependent activation. It has previously been reported that extracellular Mg^2+^ can block inward TREK-1 currents at hyperpolarized potentials (Maingret et al., 2002). However, we found that extracellular divalent cations only had minor effects on voltage activation and tail currents (Figures S2A–S2C).

### The Voltage-Dependent Gate Is Located within the Selectivity Filter

**Figure S3:**
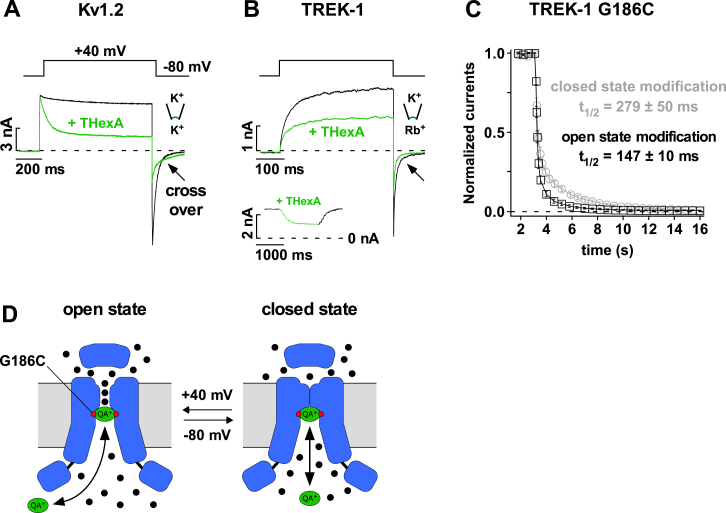
The Intracellular Pore Entrance Is Not the Voltage Gate in K2P Channels, Related to Figure 2 (A) Current responses of Kv1.2 channels to indicated voltages with and without 1 μM THexA (tetrahexylammonium) showing the typical time course of state-dependent open channel block and the slowing of deactivation resulting in a ‘cross over’ of the tail currents. (B) Same protocol as in (A) but for TREK-1 channels indicating that TREK-1 is inhibited by THexA before voltage activation and that current deactivation is not slowed by THexA. The inset shows the time course of THexA inhibition applied on voltage-activated (at +80 mV) TREK-1 channels via a rapid application system indicating that blocker binding is much slower than activation (note the different scale bars). (C) Time course of MTS-TBAO (20 μM) modification of TREK-1 G186C channels determined at +40 mV and at −80 mV (to assess the fraction of modified channels very briefly (10 ms) stepped to +40 mV). Experiments for TREK-1 channels were performed in solutions containing Rb^+^ on the intracellular side of the membrane (120 mM [Rb^+^]_int._/120 mM [K^+^]_ext._) to increase current amplitudes. (D) Cartoon depicting that the intracellular pore entrance is open and accessible to MTS-TBAO modification and THexA (QA^+^) binding in both closed and open states of TREK-1 channels. Data are represented as mean ± SEM.

The voltage-dependent gate in classical Kv channels is located at the entrance to the intracellular pore (Yellen, 1998). We therefore employed two established protocols to probe for a functional lower gate, i.e., state-dependent pore cysteine modification and state-dependent pore blocker binding (Figures S3A–S3C) (Piechotta et al., 2011, Rapedius et al., 2012). Consistent with our previous findings, chemical modification of a pore residue in TREK-1 occurs with a similar rate regardless of whether the channels are activated or closed by voltage (Figure S3C). Furthermore, we found that a high-affinity quaternary ammonium (QA^+^) pore blocker has free access to its binding site deep within the pore even when TREK-1 channels are closed at hyperpolarized potentials (Figure S3B). These results indicate that the voltage-gating mechanism likely resides within or close to the selectivity filter (Figure S1D).

### Mutation of the S4 K^+^ Binding Site Abolishes Voltage Gating

**Figure 2:**
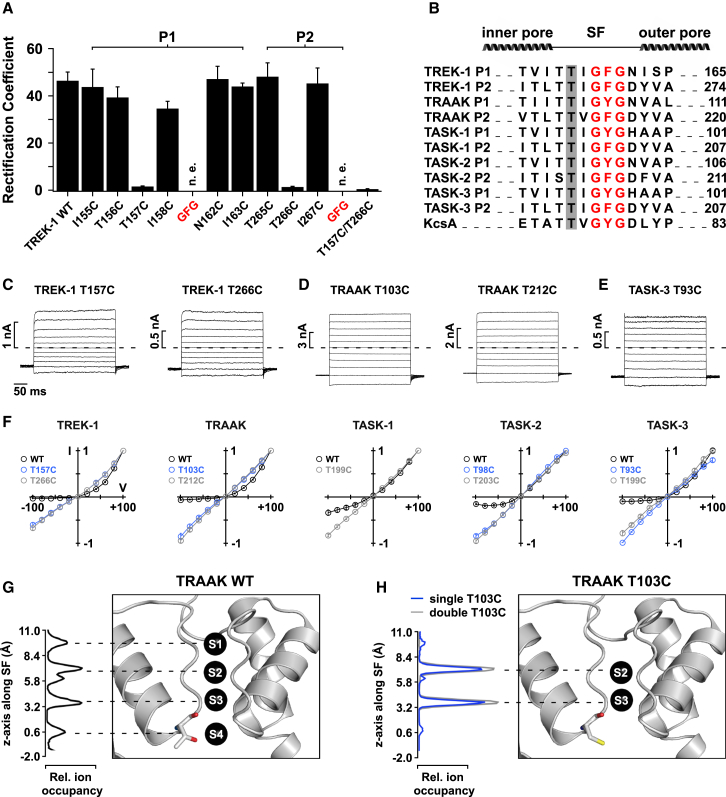
The Filter Represents the Voltage Gate in K2P Channels (A) The bars represent the rectification coefficients (for WT TREK-1 channels and indicated mutations around the GFG motif in P1 and P2. (B) Sequence alignment of the filter regions for probed K2P channels (and KcsA) is shown, and the critical threonine is highlighted. (C–E) Current responses to voltage families for mutant TREK-1 (C), TRAAK (D), and TASK-3 channels (E) show loss of voltage gating for threonine mutants. (F) I-V plots for WT and mutant channels. (G and H) MD simulations performed on TRAAK show relative ion occupancies for the ion binding sites S1–S4 (G) and their change (H) upon mutation of either one (single T103C) or both positions (double T103C), leading to a loss of binding to S1 and S4. Data are represented as mean ± SEM. See also Figure S3.

To investigate the involvement of the filter, we mutated residues within either the first (P1) or second (P2) pore loops of TREK-1 channels. As expected, mutating the conserved GFG motif of the filter resulted in non-functional channels, but all other mutants generated macroscopic currents (Figure 2A). Strikingly, two mutations completely removed this voltage-dependent gating behavior, namely, mutations of the highly conserved threonine within the consensus K^+^-selective motif (TIGFG) in both P1 and P2 (Figure 2B). These two mutants (T157C and T266C) displayed linear I-Vs and instantaneous current-voltage responses that completely lacked the prominent tail currents observed in the WT channel (Figure 2C). In contrast, the other mutations within P1 and P2 had no obvious effect on voltage gating and did not change the large rectification coefficient of 47 ± 4 (Figure 2A) that defines outward rectification in WT TREK-1. Remarkably, similar results were also obtained when the corresponding threonines (in P1 and P2) were mutated in TRAAK, TASK-1, TASK-2, and TASK-3 channels (Figures 2D–2F), suggesting these highly conserved residues are important for the voltage-gating mechanism within the K2P channel superfamily.

Intriguingly, the hydroxyl group within these threonines is the only side chain within the filter that interacts directly with K^+^ ions. The filter contains four K^+^ binding sites (S1–S4), and mutating these residues is therefore expected to change ion occupancy at these sites within the filter. Indeed, in the KcsA K^+^ channel, the equivalent mutation (T75C) reduces K^+^ binding to the S4 site, presumably because sulfur coordinates K^+^ less well than oxygen (Zhou and MacKinnon, 2004).

### MD Simulations Reveal the Filter Ion Occupancy of WT and Mutant Channels

To gain insight into the ion occupancy of the K2P channel filter, we employed atomistic molecular dynamics (MD) simulations with a double bilayer setup as recently used to simulate ion permeation in other K^+^ channels, such as KcsA and Kv1.2 (Köpfer et al., 2014). Using the crystal structure of TRAAK (Brohawn et al., 2013), 200 permeation events over a 10 μs simulation (20 events/μs) were detected under positive voltage. Analysis of these events suggests that ion permeation occurs by a similar mechanism as previously reported for KcsA (Köpfer et al., 2014). Furthermore, the average ion occupancy during permeation was 2.7 ± 0.6 and thus was similar to KcsA (Köpfer et al., 2014). The four K^+^ binding sites (S1–S4) in the filter showed about similar occupancy (S1 = 58%, S2 = 97%, S3 = 65%, and S4 = 46%; Figure 2G) in TRAAK. In silico exchange of the two threonines by cysteines at the P1 site (i.e., T103C) led to a loss of K^+^ binding to the S1 and S4 site, as well as a large reduction in permeation events (1.25 events/μs). However, introduction of a single exchange (i.e., T103C of just one of the two threonines in P1) increased the number of permeation events (11.4 events/μs), although the ion occupancy at the S1 and S4 site remained low (Figure 2H). This confirms that mutation of the S4 threonine causes a major change in filter ion occupancy similar to that reported for KcsA (Zhou and MacKinnon, 2004). Combined with our functional mutagenesis data shown above, these results suggest that the ion binding in the filter represents a critical step in this voltage-gating mechanism.

### Voltage-Dependent Gating Is Affected by the Permeant Ion Species

**Figure 3:**
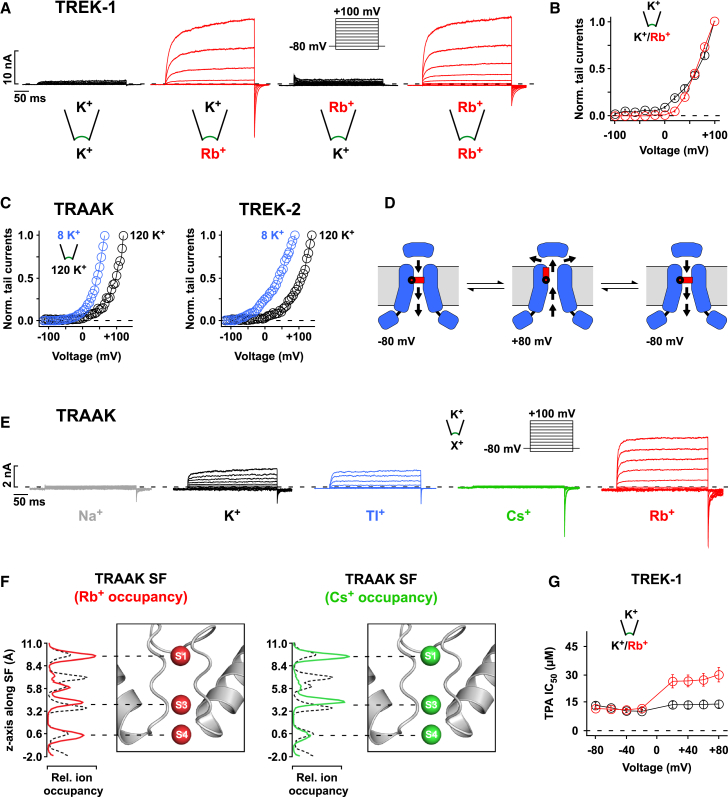
Ion-Flux Gating Is Sensitive to the Permeant Ion Species (A) TREK-1 current responses to voltage families in symmetrical K^+^ (120 mM [K^+^]_int._/120 mM [K^+^]_ext._) or with K^+^ exchanged by Rb^+^ on the intracellular, extracellular, or both sides of the membrane patches. (B) Normalized tail current amplitudes for the indicated pre-pulse potentials with 120 mM K^+^_int._ (black circles) and 120 mM Rb^+^_int._ (red circles); data are as mean ± SEM. (C) Circles represent I-V curves with 8 mM or 120 mM K^+^_ext._ for TRAAK and TREK-2 indicating the parallel shift of voltage activation and E_rev._. (D) Cartoon depicting the check-valve ion-flux gating behavior with outward ion permeation opening the selectivity filter and inward ion movement closing the filter; the structure above the pore indicates the extracellular cap domain. (E) Voltage-evoked TRAAK currents with 120 mM K^+^_ext._ and various intracellular ions (120 mM) as indicated. (F) MD simulations performed on TRAAK show the relative ion occupancies for the S1–S4 sites with Rb^+^ and Cs^+^ permeation in comparison to K^+^ permeation (black dotted line). (G) The IC_50_ for TREK-1 channel inhibition by TPA^+^ was determined for the respective voltages in symmetrical K^+^ and with intracellular Rb^+^ replacing K^+^; data are as mean ± SEM. See also Figure S4.

To further explore the role of the filter, we next investigated the ion dependence of voltage activation. Strikingly, replacement of intracellular K^+^ by Rb^+^ increased the voltage-dependent activation of TREK-1 around 100-fold, resulting in large outward currents and large tail currents (Figure 3A). The voltage dependence of the increase in tail currents (reflecting the slope of the increase in *Po*) was not altered in comparison to K^+^ (Figure 3B). Importantly, the potentiation of currents by Rb^+^ only occurred upon substitution with intracellular Rb^+^ (Figure 3A) and therefore with outward Rb^+^ permeation. These results not only suggest that Rb^+^ potentiates voltage activation but that the direction of ion-flux is also critical, i.e., K2P channels appear to be gated by a one-way, non-return, or “check valve”-like ion-flux mechanism through which outward movement of ions gates the filter open, while inward permeation gates the filter closed (Figure 3D). In agreement with this, we found that changing the reversal potential by altering the extracellular K^+^ concentration caused a parallel shift in the voltage dependence of activation for TRAAK, TREK-2, and TASK-3 (Figures 3C and S5C).

**Figure S4:**
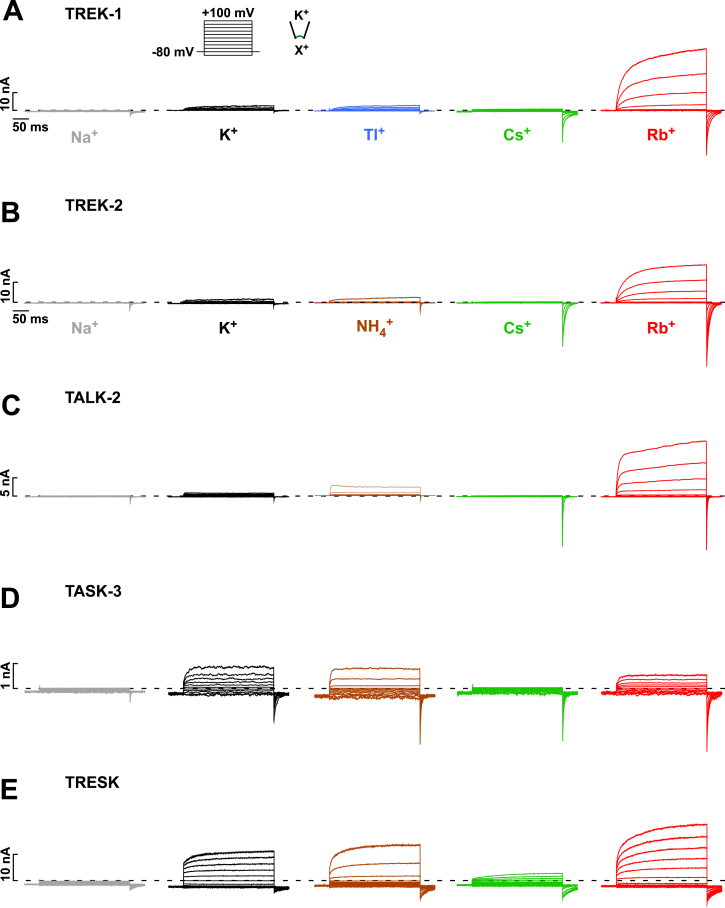
Ion Activation Profiles for Voltage Activation in Various K2P Channels, Related to Figure 3 (A–E) Depicted voltage family evoked currents with 120 mM [K^+^]_ext._ and various intracellular ions (120 mM [Na^+^], [Tl^+^], [Cs^+^], [NH_4_^+^] and [Rb^+^]_int._) obtained in same patches for: (A) TREK-1, (B) TREK-2, (C) TALK-2, (D) TASK-3, (E) TRESK.

We also measured voltage activation for different K2P channels (TRAAK, TREK-1, TREK-2, TALK-2, TASK-3, and TRESK) with various intracellular permeant ions (Figures 3E and S4A–S4E). In TRAAK we found that, similar to TREK-1, voltage activation was potentiated by Rb^+^. Furthermore, although Cs^+^ was poorly permeable (producing only small outward currents), it evoked large tail currents for potentials positive to the reversal potential. Importantly, this demonstrates that it is the ion binding step within the filter and not the permeating rate that determines voltage activation. With intracellular Na^+^ the tail currents were small in all tested K2P channels (Figures 3E and S4A–S4E), as were the tail currents using intracellular NMDG^+^ (data not shown). In TASK-3 and TRESK channels only a small potentiation by intracellular Rb^+^ or Cs^+^ was observed (Figures S4D and S4E).

For TRAAK and TREK-1 channels, we found that intracellular Tl^+^ behaved similar to K^+^, i.e., its effects were markedly less pronounced than those of Cs^+^ or Rb^+^ (Figures 3E and S4A). Intriguingly, this ionic profile for voltage gating correlates with differences in ion occupancies observed crystallographically within the filter of KcsA. In these structures, K^+^ and Tl^+^ bind with similar occupancy to all four sites (S1–S4), whereas Cs^+^ and Rb^+^ bind with a different profile to only three sites that overlap predominantly with S1, S3, and S4 (Zhou and MacKinnon, 2003). To investigate the relative occupancy of these ions in a K2P channel, we simulated permeation in TRAAK with Rb^+^ and Cs^+^. These simulations clearly show a different distribution compared to K^+^ (Figure 3F), with preferential occupancy of S1, S3, and S4 by Cs^+^ and Rb^+^. Binding of these ions to the S2 site was strongly reduced. Furthermore, the reduction in occupancy of the S2 site caused a marked increase in S4 occupancy for Rb^+^ compared to K^+^ (Figure 3F).

Further evidence for the effect of permeant ions on the filter comes from studies of blockers, such as tetrapentylammonium (TPA^+^), that interact with the threonine side chains of the S4 site and should therefore directly compete with ions for binding to this site (Piechotta et al., 2011). We reasoned that upon switching from K^+^ to Rb^+^, TPA^+^ affinity may drop as a result of increased occupancy of S4 by Rb^+^ and thus increase competition with TPA^+^. Indeed, with intracellular Rb^+^ (and extracellular K^+^) we found that TPA^+^ affinity dropped at the point at which permeation switches from K^+^ to Rb^+^, i.e., at voltages positive to the E_rev._ (Figure 3G). However, in symmetrical K^+^ voltage dependence of TPA^+^ inhibition was neither seen upon current inversion nor was TPA^+^ affinity voltage dependent when the filter conducted either Rb^+^ or K^+^ (Figure 3G). This provides direct functional validation of the MD simulations and suggests that Rb^+^ potentiation of voltage activation results from a change in filter ion occupancy.

### Measuring the Gating Charge

**Figure 4:**
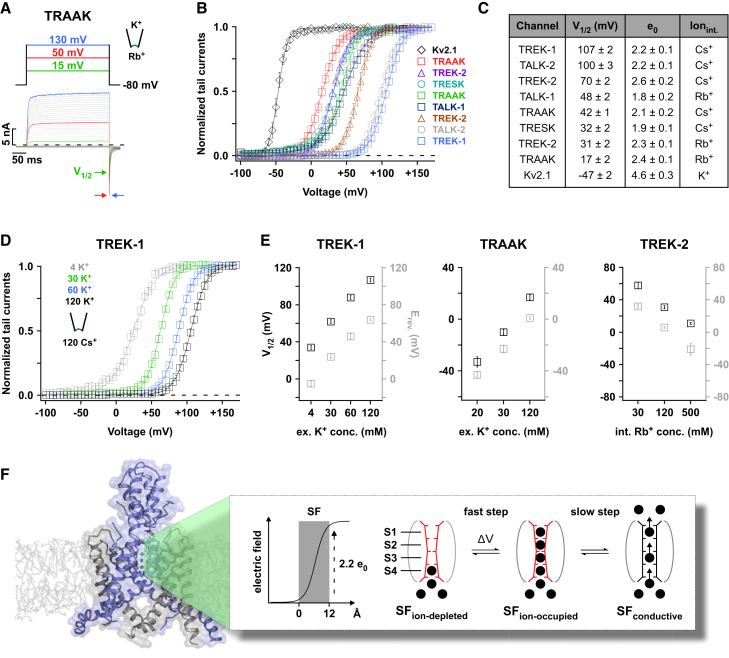
Voltage Sensitivity Arises from the Movement of Ions within the Electric Field of the Selectivity Filter (A) TRAAK currents with intracellular Rb^+^ (120 mM [Rb^+^]_int._/120 mM [K^+^]_ext._) for different potentials show a maximal *P*_*O*_ achieved for potentials positive to +50 mV as further depolarization does not increase tail current amplitudes. (B) Normalized tail current versus pre-pulse voltage plots for the indicated K2P channels and intracellular ions. (C) Tail current pre-pulse voltage relations as in (B) fitted to a standard Boltzmann function. The table shows the fit parameters V_1/2_ ± SEM and z (= equivalent gating charge [e_0_]) ± SEM for the indicated K2P channels and intracellular ions. (D) The V_1/2_ of voltage activation shifts with E_rev._ as shown here for tail current amplitude versus pre-pulse voltage plots for TREK-1 with different K^+^_ext._. (E) V_1/2_ of voltage activation (black squares) and measured E_rev._ values (gray squares) are plotted for TREK-1, TREK-2, and TRAAK channels with various extracellular and intracellular ion concentrations. See also Figures S5A and S5B. (F) Cartoon depicting the voltage sensor in K2P channels. The structure represents TRAAK (adopted from Brohawn et al., 2012) embedded in a bilayer; the electric potential drop across the selectivity filter (SF) is indicated. The inactivated SF is shown as a structurally distinct ion-depleted state (SF_ion-depleted_). Upon depolarization, 3–4 ions are forced into the filter by the high-electric field (SF_ion-occupied_). This highly charged state is not stable and then transforms into the permeating state (SF_conductive_). See also Figure S5.

In Kv channels, the gating charge that is coupled to pore opening is determined by plotting the tail current amplitudes (i.e., the relative increase in *P*_*O*_) against the activating pre-pulse voltages. The slope of a standard Boltzmann fit to the derived G-V plots represents the equivalent gating charge and thus the fraction of energy used to open the channel when charge is translocated across the entire electric field of the membrane. For comparison, we determined the equivalent gating charge for Kv2.1 channels and, similar to previous measurements (Scholle et al., 2004), found it to be 4.6 ± 0.3 elementary charges (e_0_) (Figures 4B and 4C).

To fit the G-V plots of K2P channels with a Boltzmann function requires the tail current amplitudes to saturate with a change in voltage. For K^+^ (and Tl^+^), no saturation was seen. However, for Rb^+^ and Cs^+^ saturating G-V curves were observed revealing an equivalent gating charge between 1.8 and 2.6 e_0_ for all the K2P channels tested (TRAAK, TREK-1, TREK-2, TALK-1, TALK-2, and TRESK; Figures 4A–4C). When averaged for all these channels, the gating charge was about 2.2 e_0_. This value represents the lower limit of charge movement coupled to the channel opening reaction (Sigg and Bezanilla, 1997).

If we assume the membrane electric field is focused on the filter (Contreras et al., 2010, Jiang et al., 2002), then a charge equivalent to at least 2.2 e_0_ must be translocated across the filter to convert it into the open conformation (Figure 4F). For this to occur, we must assume that the inactive filter (i.e., at −80 mV) represents an “ion-depleted” state containing only one (or zero) ions and may therefore be similar to the “C-type-inactivated” state observed in KcsA (Boiteux and Bernèche, 2011, Cuello et al., 2010). Our model then predicts that upon depolarization, 3–4 additional ions are forced into the filter by the electric field and that this ion translocation step is reflected in our measurement of the equivalent gating charge (Figure 4F). Furthermore, because the time course of activation is voltage independent, a second gating step must follow this ion translocation step, and this is represented by the slower, time-dependent conformational change of the filter into the active (i.e., conductive) state (Figure 4F).

### Voltage Activation Is Tightly Coupled to the Electrochemical Gradient

**Figure S5:**
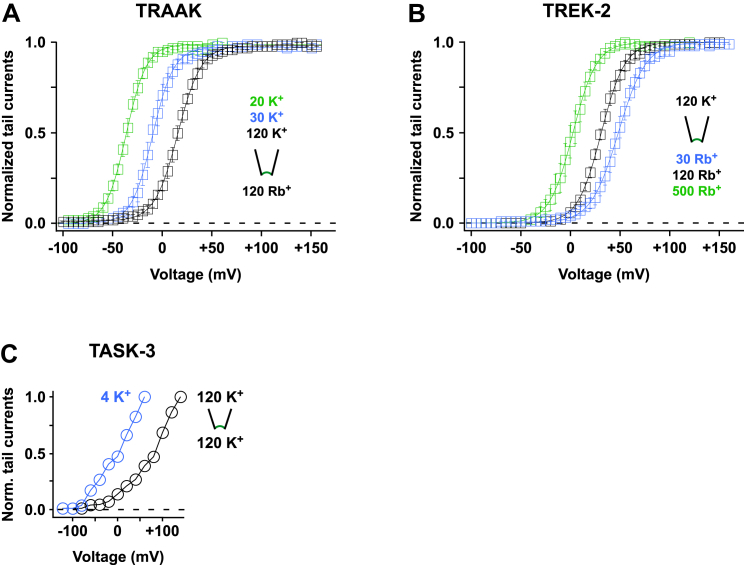
The V_1/2_ of Voltage Activation Shifts with the E_rev._, Related to Figure 4 (A) Normalized tail current amplitudes for TRAAK measured with 120 mM [Rb^+^]_int._ and different extracellular K^+^ concentrations. (B) Normalized tail current amplitudes for TREK-2 channels measured with 120 mM [K^+^]_ext._ and different intracellular Rb^+^ concentrations. (C) Normalized tail current amplitudes for TASK-3 channels measured in symmetrical K^+^ (120 mM [K^+^]_int._/120 mM [K^+^]_ext._; black circles) and with low extracellular K^+^ (120 mM [K^+^]_int._/4 mM [K^+^]_ext._; blue circles). Data are represented as mean ± SEM.

To further validate this model, we investigated the relationship between the direction of ion flow and voltage activation by measuring the V_1/2_ value at varying extracellular K^+^ concentrations for several different K2P channels (TRAAK, TREK-1, and TREK-2). We found that V_1/2_ shifted in parallel with the reversal potential (i.e., the voltage point at which ion-flux reverses), regardless of whether the intracellular or extracellular ion concentration was changed (Figures 4D, 4E, S5A, and S5B). This clearly demonstrates that it is not the transmembrane voltage per se that drives K2P channel gating, but the electrochemical driving force, i.e., the difference (Δμ) between the actual membrane voltage (V_m_) and the reversal potential (E_rev._), (Δμ = V_m_ – E_rev._).

### K2P Channel Activation Causes a Gating Mode Shift within the Selectivity Filter

**Figure 5:**
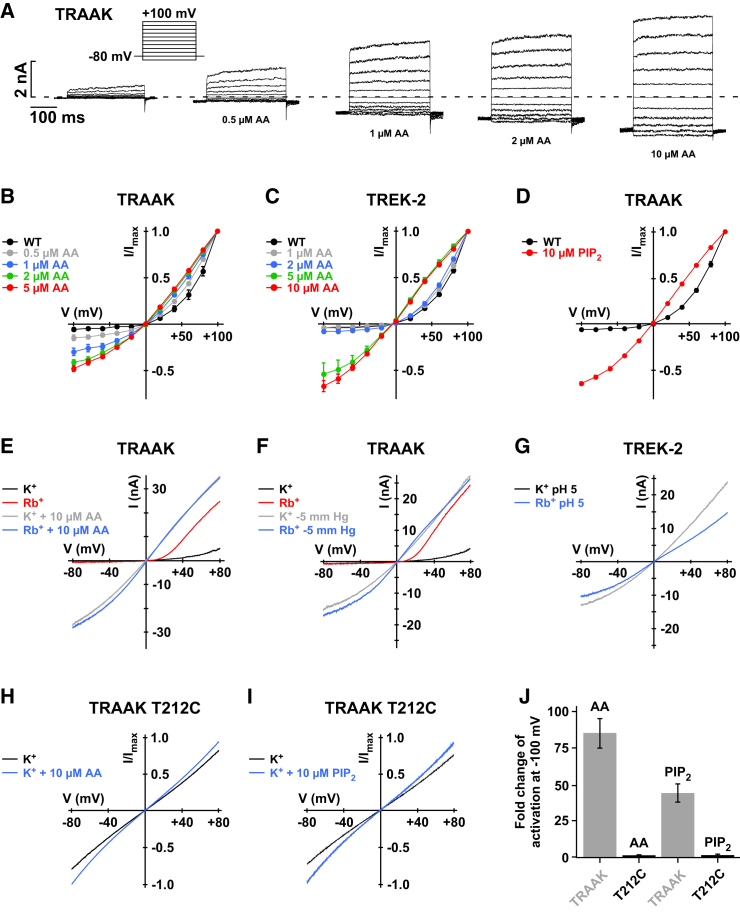
K2P Channel Activators Switch the Filter Gating Mode (A) TRAAK currents recorded with increasing arachidonic acid (AA) concentrations in symmetrical K^+^ from inside-out patches. (B and C) From experiments as shown in (A), I-V curves are plotted for different AA concentrations for TRAAK channels (B) and TREK-2 channels (C). (D) I-V plots for TRAAK currents before and after channel activation by 10 μM PIP_2_. (E) Representative ramp measurements of TRAAK channels activated by 10 μM AA with either K^+^ or Rb^+^ as the intracellular ion. (F) Representative ramps of TRAAK channels activated by 5 mmHg negative pressure applied via the patch pipette with either K^+^ or Rb^+^ as the intracellular ion. (G) Representative ramps of TREK-2 activated by pH_int._ 5.0 with either K^+^ or Rb^+^ as the intracellular ion. (H and I) TRAAK T212C currents lack activation by AA and PIP_2_. (J) Fold current change (current_(+activator)_/current_(–activator)_) for TRAAK WT and TRAAK T212C channels upon application of 10 μM AA or 10 μM PIP_2_ at −100 mV; data are as mean ± SEM. See also Figure S6.

**Figure S6:**
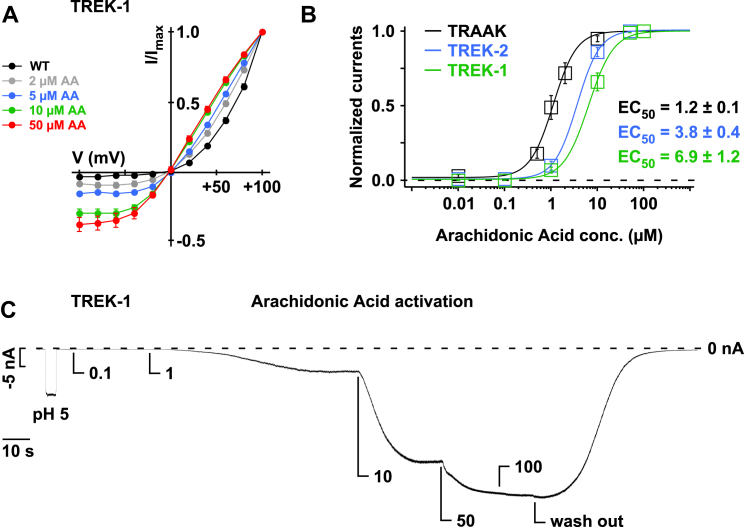
Arachidonic Acid Activation in TRAAK, TREK-1, and TREK-2 Channels, Related to Figure 5 (A) I-V plots obtained for TREK-1 channels in the absence and the presence of different AA concentrations. (B) Dose-response curves for AA activation of different K2P channels. EC_50_ values of standard Hill fits are indicated. (C) TREK-1 current at −80 mV upon application of pH_int._ 5 and increasing AA concentrations as indicated. Data are represented as mean ± SEM.

TRAAK, TREK-1, and TREK-2 channels are activated by arachidonic acid (AA) (Honoré, 2007). We therefore investigated how AA affects voltage gating by obtaining I-V curves in a symmetrical K^+^ gradient in which voltage gating is most obvious. In inside-out patches, AA activated TRAAK, TREK-1, and TREK-2 channels with EC_50_ values of 1.2 ± 0.1, 6.9 ± 1.2, and 3.8 ± 0.4 μM, respectively (Figures S6B and S6C). We also found that this strong outwardly rectifying behavior progressively transformed into a linear conductance with increasing concentrations of AA (Figures 5A–5C and S6A). For AA concentrations below the EC_50_ values, outward rectification and the corresponding tail currents were still prominent, but at higher concentrations this effect almost completely disappeared (Figures 5A to 5C and S6A).

To examine whether other regulatory mechanisms affect this process, we activated these channels with pressure, PIP_2_, and also acidic pH and found that all of these stimuli reduced voltage activation and resulted in linear I-Vs similar to AA activation. Furthermore, activation of TRAAK also abolished Rb^+^ potentiation of voltage activation (Figures 5E and 5F). Moreover, the T212C filter mutation that abolished voltage activation in TRAAK channels (Figure 2D) also removed activation by AA and PIP_2_ (Figures 5H–5J). This provides further evidence that these different mechanisms converge on the filter as the primary gate.

### Voltage Activation of K2P Channels under Physiological K^+^ Conditions

**Figure 6:**
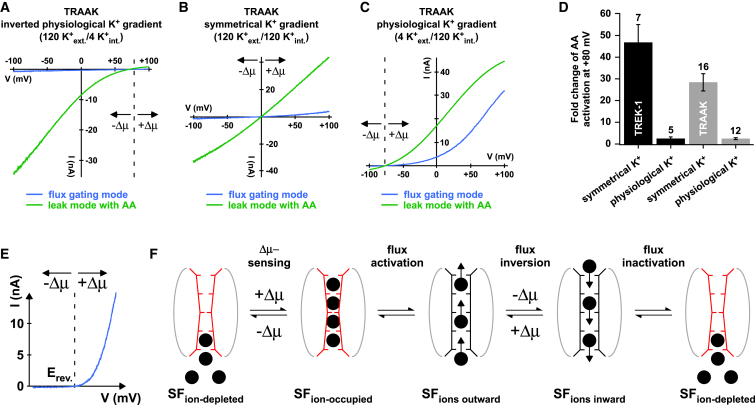
The Selectivity Filter Senses the Electrochemical Potential (A–C) TRAAK current responses with an inverted physiological K^+^ gradient (4 mM K^+^_int._/120 mM K^+^_ext._) (A), symmetrical K^+^ gradient (120 mM K^+^_int._/120 mM K^+^_ext._) (B), and physiological K^+^ gradient (120 mM K^+^_int._/4 mM K^+^_ext._) (C) before and after AA (10 μM) activation. (D) Bars (mean ± SEM) indicate fold increase of currents at +80 mV upon AA activation for a physiological gradient compared to a symmetrical gradient in TREK-1 and TRAAK channels. (E) Illustration of the electrochemical driving force (i.e., Δμ = V_m_ − E_rev._) dependence of K2P gating with a positive Δμ, leading to activation but inactivation for a negative Δμ. (F) Cartoon of the proposed mechanism of flux-coupled gating depicting that for a negative Δμ (i.e., all voltages negative to the E_rev._) the filter is ion-depleted and inactive (SF_ion-depleted_). For voltages positive to E_rev._, the channels start to activate as an increasing fraction of the channels populate the inactive but now ion-occupied state (SF_ion-occupied_) that rapidly converts into the active outwardly permeating state (SF_ions-outward_). Upon inversion of the driving force (i.e., for potentials negative to the E_rev._), the SF only transiently conducts inward currents as this state is not stable (SF_ions-inward_) and finally adopts the initial, structurally distinct, ion-depleted, and inactive state (SF_ion-depleted_).

To further investigate K2P channel activation under physiological conditions and emphasize the importance of this ion-flux gating mechanism, we recorded TRAAK channels with a physiological K^+^ gradient (120 mM K^+^_int._/4 mM K^+^_ext._), a symmetrical K^+^ gradient (see also Figure 5A), and an inverted physiological K^+^ gradient (4 mM K^+^_int._/120 mM K^+^_ext._) (Figures 6A–6C). In an inverted K^+^ gradient, only very small currents were seen and AA produced around 100-fold increase of the inward current (Figure 6A). According to our model, the “check valve” mechanism will hold the filter closed for inward currents, and only when the valve is opened by AA will large inward currents be able to develop. With a symmetrical K^+^ gradient, a large increase of inward current is also seen (Figure 6B). The fold increase upon AA stimulation for the outward currents was smaller consistent with the finding that flux gating activates TRAAK to some degree at depolarized potentials (Figures 5A and 6B). By marked contrast, when a physiological K^+^ gradient is used, large outward currents are seen due to the large outwardly directed chemical driving force that pushes a significant fraction of channels into the open filter state (6C). In this situation, AA only induces a relatively modest increase (2.6- ± 0.5-fold) in current at strongly depolarizing potentials (e.g., +80 mV) because the ion-flux mechanism has already opened the filter (Figures 6C and 6D). Similar results were obtained for TREK-1 (Figure 6D). These results therefore demonstrate that under physiological conditions (i.e., low K^+^_ext._) unstimulated TRAAK and TREK channels also generate significant outward currents upon depolarization because the negative E_rev._ strongly favors the ion-flux gating mechanism at these potentials.

## Discussion

In this study we reveal how an ion channel can achieve unprecedented sensitivity to changes in transmembrane voltage without a canonical VSD and have resolved the mechanisms underlying this effect. We also show that this voltage sensitivity is common to nearly all K2P channels, thereby redefining our understanding of this family.

### Voltage Activation versus GHK-type “Leak” Currents

Our findings demonstrate that voltage sensing in K2P channels results from an ion-flux gating mechanism directly powered by the electrochemical K^+^ gradient. Although this produces strong outward rectification, we show that it is distinct from the non-linear K^+^-selective “leak” behavior of an open pore in an asymmetric (i.e., physiological) K^+^ gradient defined by the Goldman-Hodgkin-Katz (GHK) equation. We therefore believe that the term “ion-flux-gated” instead of “leak” may be more appropriate to describe the intrinsic biophysical properties of most K2P channels.

However, some K2P channels (in particular TRAAK, TREK-1, and TREK-2) can be converted into a “classical” GHK-leak mode when stimulated by, e.g., AA or PIP_2_. Nevertheless, under physiological conditions, flux gating will still be relevant because the negative E_rev._ strongly promotes voltage activation at depolarized potentials as would occur during an action potential. Furthermore, for K2P channels that are not activated by such stimuli (e.g., TASK channels) the flux gating mode may be the prevalent gating mechanism.

Ironically, the TWIK-1 channel, after which all other K2P channels were originally named (Lesage et al., 1996), appears to be the only K2P channel that is genuinely a K^+^-selective GHK-type “leak” channel. This is possibly related to the unusual structural and functional properties of the selectivity filter in TWIK-1 (Chen et al., 2014) because we show that changes in the filter sequence of other K2P channels can abolish flux gating.

### Gating Charge Consists of Three to Four Ions Moving into the Filter

For the voltage-sensing step, we determined an average equivalent gating charge of 2.2 e_0_ and the inactive filter as the voltage-sensing motif. To achieve such a gating charge, we propose that the inactive filter must exist in either an “ion-depleted” state (with only one or zero ions) or a fully “ion-occupied” state. The relative distribution of these two states can then be described by a voltage-dependent Boltzmann function.

The voltage drop along the 12 Å distance of the filter has been estimated to represent ∼80% of the drop across the entire thickness of the membrane (Figure 4F) (Contreras et al., 2010, Jiang et al., 2002). Consequently, if this voltage drop occurs linearly within the filter then translocation of the first ion from the inner S5 cavity site (outside the electric field) to the extracellular S1 site would contribute 0.8 e_0_, translocation of a second ion to S2 would contribute about 0.6 e_0_ (i.e., three-fourths of 0.8 e_0_), translocation of a third ion to S3 about 0.4 e_0_ (one-half of 0.8 e_0_) and a fourth ion to S4 about 0.2 e_0_ (one-fourth of 0.8 e_0_). These charges sum to 2.0 e_0_, thus closely matching the value we have determined experimentally. Although this is only a rough estimation, it strongly suggests that the inactive filter must be almost completely devoid of ions at negative potentials, but fully occupied at depolarized potentials. It also indicates that three to four ions are forced into the electric field of the filter at potentials positive to the E_rev._. To the best of our knowledge, this represents the first direct electrophysiological measurement of the number of ions that can simultaneous occupy the filter in a K^+^ channel. Previously, such high ion occupancy was considered unlikely due to electrostatic repulsion (Doyle et al., 1998, Zhou and MacKinnon, 2003). However, recent MD simulations and re-evaluation of crystallographic data have questioned this view to suggest that four ions can simultaneously occupy the filter (Köpfer et al., 2014). Our experimental data are in good agreement with this idea.

### The Filter Senses Both Voltage and Chemical Gradients

For simplicity, we have, until now, mainly considered changes in voltage with a constant (i.e., symmetrical) K^+^ gradient across the membrane. However, we now show that voltage gating is directly coupled to the E_rev._ (e.g., Figure 4E). Furthermore, this coupling occurs independently of how E_rev._ is changed (i.e., by changing either the intracellular or extracellular ion concentration) thereby suggesting that filter ion occupancy is a direct function of both the voltage and chemical gradients. This indicates that the inactive filter conformation senses the electrochemical driving force; i.e., it changes its ion occupancy according to the electrochemical potential. For this to happen, we propose that the “inactive” filter is not entirely impermeable, but instead conducts ions at a very low rate to allow continuous equilibration of ion binding according to the actual electrochemical potential.

### A One-Way “Check Valve”-like Ion-Flux Gating Model

The gating scheme we propose in Figure 6 provides an explanation for the “check valve” ion-flux gating mechanism. In this model the filter can adopt at least four distinct states: two inactive states (an “ion-depleted” inactive state and an “ion-occupied” inactive state), plus two active states of the filter that also differ in ion occupancy. Therefore, when exposed to strong negative electrochemical driving forces (i.e., when V_m_ is negative to E_rev._ and Δμ < < 0; Figure 6E) the filter will exist in an “ion-depleted” inactive state and depolarization (leading to a positive Δμ) will force several ions into the filter reflecting the measured gating charge.

Subsequent to this presumably very rapid ion translocation step the channel will still reside in an inactive, but “ion-occupied” state that is now capable of transformation into a highly active (i.e., conductive) state, and it is this structural transition that reflects the voltage-independent kinetics of channel activation. It is possible that the high degree of ion occupancy within the filter and direct coulombic interactions between the ions may exert sufficient forces on the filter to power this opening reaction. However, it is clear that upon a sudden inversion of this driving force (i.e., repolarization) the stability of this activated state rapidly decreases, resulting in inactivation of the large inward (tail) currents. We propose that the channel then reverts to a structurally distinct “ion-depleted” inactive state (see cartoon in Figure 6F).

Central to this mechanism is that the active filter becomes unstable when it conducts inward current. A straightforward explanation is that filter ion occupancy depends on the direction of ion-flux. We therefore speculate this may result from different probabilities of occupancy of the S1–S4 sites during inward and outward permeation. Indeed, reducing K^+^ binding to S1 and S4 (e.g., with filter mutations in TRAAK) prevents inactivation of this inward flux. Further simulation studies using the computational electrophysiology method we describe here may be helpful to dissecting this process in more detail.

### Relationship to Other Mechanisms of Voltage Sensing

In classical voltage-gated K^+^ channels the mechanisms of voltage sensing, activation, and inactivation are assigned to different regions of the protein, i.e., the voltage-sensing domains, the helix-bundle crossing gate, and the selectivity filter gate, respectively. By contrast, K2P channels appear to have all of these different functional properties condensed within alternative structural states of the filter (Figure 6F).

Interestingly, TOK1, a yeast 8-TM K2P channel, displays gating properties similar to those described here for the mammalian K2P channels, including an extracellular K^+^ dependence (Ketchum et al., 1995, Loukin and Saimi, 1999). However, no tail currents have been reported for TOK1. It will therefore be interesting to determine whether mutations in the filter and/or the permeating ion species affect voltage gating in these unusual K2P channels.

Voltage gating mediated by the selectivity filter has also been reported in other ion channels. However, the precise mechanisms appear different and the voltage dependence is clearly not as marked as in K2P channels. In KcsA an equivalent gating charge of 0.7 e_0_ results from reorientation of a charged residue within the filter (Cordero-Morales et al., 2006). In MthK a voltage- and extracellular ion-dependent inactivation mechanism results from the depletion of K^+^ from the filter by strong depolarization (Thomson and Rothberg, 2010).

However, the most similarity may be with the voltage-gating properties of CLC Cl^−^ channels (Miller, 2006). These channels also lack an obvious voltage-sensing domain, but display voltage gating with an equivalent gating charge of ∼1 e_0_ and a strong anion dependency. This is thought to arise from Cl^−^ ions acting as the gating charge and displacing a critical glutamate residue in the pore that otherwise blocks ion permeation (De Jesus-Perez et al., 2016, Miller, 2006, Dutzler et al., 2003). However, voltage-dependent movement of H^+^ and protonation of this glutamate have also been suggested to contribute to the voltage dependence (Pusch, 2004). The H^+^ dependency might also be related to the evolutionary link between CLC Cl^−^ channels and CLC Cl^−^/H^+^ transporters (Miller, 2006). Interestingly, although K2P channels bear no structural resemblance to transporters, this flux gating mechanism further closes the mechanistic gap between ion channels and transporters as both mechanisms utilize the electrochemical gradient to power a conformational change, one being an ion translocation step (transporter) and the other a pore gating step (K2P channel).

In a broader sense, many examples have now been reported in which the permeating ion species can have a marked effect on channel gating, including BK, Kv, and Kir channels (Baukrowitz and Yellen, 1996, Dahlmann et al., 2004, Demo and Yellen, 1992, Seebohm et al., 2003). Of particular relevance is the mechanism of C-type inactivation studied in KcsA, where the link between filter ion occupancy and inactivation has been resolved in great detail (Cuello et al., 2010). Interestingly, C-type inactivation in KcsA appears to require ion occupancy of the S2 site and Rb^+^ reduces this inactivation, presumably due to its lack of binding at S2 (Matulef et al., 2013). Intriguingly, our MD simulations with TRAAK also show a reduced occupancy of S2 by Rb^+^ accompanied by dramatic stabilization of the open state that might also be interpreted as reduced filter inactivation. Therefore, S2 ion occupancy appears critical for both ion-flux gating in K2P channels and C-type inactivation, suggesting they may involve similar structural changes in the filter and, possibly also, in the transmembrane domains as reported for KcsA (Cuello et al., 2010).

### K2P Channel Activation Alters the Gating Properties of the Filter

The mechano-gated subfamily of K2P channels (TRAAK, TREK-1, and TREK-2) are activated by a wide range of stimuli (Honoré, 2007). Furthermore, activation by PIP_2_, intracellular pH and membrane stretch have been shown to abolish voltage activation in TREK-1 (Chemin et al., 2005), thus effectively causing a gating mode shift. A similar shift from the voltage-gated mode to the leak mode has also been reported to occur upon dephosphorylation of TREK-1 channels (Bockenhauer et al., 2001). We now show that these conversions result from a mode shift in the gating properties of the filter. Our results also suggest that K2P agonists act by stabilizing the activated state of the filter, thereby shifting the equilibrium away from the voltage-sensitive inactive state. The structural basis of this mode conversion remains unknown, but PIP_2_ and pH act on the C terminus connected to TM4 and may therefore control the filter gate via movement of TM4 (Bagriantsev et al., 2011, Lolicato et al., 2014, Piechotta et al., 2011).

### Physiological Implications

The strong dependence of channel activity on the electrochemical K^+^ gradient implies that unless activated by known stimuli, flux-gated K2P channels are mostly inactive at the RMP. It might therefore be assumed they have less influence on stabilizing the RMP compared to Kir channels. However, the flux-gated mode will be of particular importance for the repolarization phase of the action potential. Furthermore, because the gating kinetics are rapid (similar to many Kv channels), they may contribute to both cardiac and fast neuronal action potentials. This property has already been implicated in supporting high-frequency action potentials in neurons, such as cerebellar granule cells, that express TASK-3 channels (Brickley et al., 2001, Brickley et al., 2007). However, the contribution of flux gating to these processes will require further investigation in native preparations.

Another distinct feature is their tight coupling to the reversal potential because this predicts different effects on changes in extracellular K^+^; a reduction will activate flux-gated K2P channels because of the positive driving force (Δμ > 0), whereas K^+^ accumulation will silence them (Δμ < 0). This is in direct contrast to Kir channels, which are activated by K^+^ accumulation (Filosa et al., 2006).

### Future Outlook

Our findings now present several exciting opportunities because they establish an experimental protocol to directly measure ion occupancy within the filter and link changes in ion occupancy to distinct gating events (i.e., voltage sensing, flux activation, and flux inactivation). Co-crystallization studies with different ions (e.g., K^+^/Tl^+^ versus Rb^+^/Cs^+^) will hopefully provide important insights into the structural changes underlying these events. It also remains unclear why the K2P channel filter produces this flux gating behavior when it has not so far been seen in other K^+^ channel. The answer likely resides outside the conserved segments of the filter and may involve its asymmetrical nature and/or the unique structural characteristics of the K2P channel pore.

## Experimental Procedures

### Molecular Biology

Human TWIK-1 (GenBank accession number:NM_002245), human TRAAK (NM_033310), rat TREK-1 (NM_172042), human TREK-2 (NM_021161), human TASK-1 (NM_002246), human TASK-2 (NM_003740), human TASK-3 (NM_001282534), human TALK-1 (NM_032115), human TALK-2 (EU978944), human TRESK (NM_181840), human Kv1.2 (NM_004974), and human Kv2.1 (NM_004975) were used in this study. Oligonucleotide-based site-directed mutagenesis was verified by sequencing, and all constructs were subcloned into the pBF, pFAW, or pSGEM expression vectors for oocyte expression. mRNAs were synthesized using the SP6 or T7 mMESSAGE mMACHINE Transcription Kit (Ambion) and stored in stock solutions at −80°C. *Xenopus* oocytes were surgically removed from adult females and treated with type II collagenase prior to manual defolliculation. About 50 nl of a solution containing channel-specific mRNA was injected into oocytes and incubated at 17°C for 1–7 days prior to use.

### Electrophysiology

Giant patch recordings in inside-out configuration under voltage-clamp conditions were made at room temperature. Pipettes were made from thick-walled borosilicate glass, had resistances of 0.3–0.9 MΩ (tip diameter of 5–15 μm), and filled with a standard pipette solution (in mM): 120 KCl, 10 HEPES, and 3.6 CaCl_2_ (pH 7.4 adjusted with KOH/HCl). Further pipette solutions contained either (in mM) 3.6 MgCl_2_ or 2 EDTA instead of 3.6 CaCl_2_. Currents were recorded with an EPC10 amplifier (HEKA electronics) and sampled at 10 kHz or higher if required and filtered with 3 kHz (−3 dB) or higher as appropriate for sampling rate. Solutions were applied via a multi-barrel pipette system to the cytoplasmic side of excised patches for the various ion channels. The standard intracellular (bath) solution had the following composition (in mM): 120 KCl, 10 HEPES, 2 EGTA, and 1 Pyrophosphate (various pHs adjusted with KOH/HCl). In other solutions K^+^ was replaced by Na^+^, NMDG^+^, Tl^+^, NH_4_^+^, Cs^+^, or Rb^+^. In some experiments K^+^, Cs^+^, or Rb^+^ was partially substituted by NMDG^+^. Appropriate pH was adjusted with hydroxide of the relevant ion species. Tetrapentylammonium chloride (TPA-Cl), tetrahexylammonium chloride (THexA-Cl), 8-(tributylammonium)octyl methanethiosulfonate bromide (MTS-TBAO-Br), arachidonic acid (AA), and phosphatidylinositol-4,5-bisphosphate (PIP_2_) were purchased from Sigma-Aldrich. All substances were stored as stocks (10–100 mM) at −80°C and diluted in bath solution to final concentrations prior to measurements. Saturating tail current amplitudes were fitted to a standard Boltzmann equation: f(x) = (amplitude/(1+exp(-(x-x_1/2_)/slope)))+offset; with the slope being RT/zF (R, universal gas constant; T, temperature; z, equivalent gating charge [e_0_]; F, Faraday constant).

### MD Simulations

MD simulations were performed as previously described (Köpfer et al., 2014) and detailed in the Supplemental Experimental Procedures.

## Author Contributions

M.S., E.N.-A., H.S., M.M., S.C., G.B., and M.R. performed all experiments and analysis supervised by B.L.d.G., S.J.T., and T.B. The manuscript was written by S.J.T. and T.B. with contributions from all authors. M.S. and E.N.-A. contributed equally to this study.

## Supplemental Information

Document S1. Supplemental Experimental Procedures

Document S2. Article plus Supplemental Information
