# Supplementary material for: A Non-canonical Voltage-Sensing Mechanism Controls Gating in K2P K+ Channels
Source: Cell. 2016 Feb 25;164(5):937–49. doi: 10.1016/j.cell.2016.02.002 (PMC4771873; doi:10.1016/j.cell.2016.02.002)
Supplement: Document S1. Supplemental Experimental Procedures [file mmc1.pdf]

Cell, Volume *164*

Supplemental Information

A Non-canonical Voltage-Sensing Mechanism

Controls Gating in K2P K^+ Channels

Marcus Schewe, Ehsan Nematian-Ardestani, Han Sun, Marianne Musinszki, Sönke Cordeiro, Giovanna Bucci, Bert L. de Groot, Stephen J. Tucker, Markus Rapedius, and Thomas Baukrowitz

Supplemental Experimental Procedures

Molecular Dynamics Simulations

MD simulations were performed using a modified version of GROMACS 4.6 (Hess et al., 2008). We used the AMBER99sb force field (Hornak et al., 2006) and SPC/E water model for the equilibrium and production simulations. Parameters for ions and lipids are derived from (Joung and Cheatham, 2008; Berger et al., 1997). Short-range electrostatic interactions were calculated with a cutoff of 1.0 nm, whereas long-range electrostatic interactions were treated by the particle mesh Ewald method (Darden et al., 1993; Essmann et al., 1995). The cutoff for van der Waals interactions was set to 1 nm. The simulations were performed at 300 K with a velocity rescaling thermostat (Bussi et al., 2007). The pressure was kept at 1 bar by means of a semi-isotropic Berendsen barostat (Berendsen et al., 1984). All bonds were constrained with the LINCS algorithm (Hess et al., 1997). Using virtual sites for hydrogen atoms allowed simulations to be performed with a 4 fs integration time step (Feenstra et al., 1999). Crystallographic structures of TRAAK including closed and open membrane opening conformations (PDB ID code: 4I9W (Brohawn et al., 2013)) were adopted to generate the initial configuration in the MD simulations. Missing atoms and loops in the crystal structure were modeled using the program loopy (Sato et al., 2008). The dimeric protein without the antibody antigen-binding fragments was embedded into an aqueous POPC lipid bilayer with 0.6 M KCl using the GROMACS tool g_membed (Wolf et al., 2010). The system was equilibrated for 20 ns with position restraints on all heavy atoms using a force constant of 1000 kJ mol^{-1} nm^{-2} to the reference structure, followed by an additional 10 ns simulation without position restraints.

For the computational electrophysiology study (Kutzner et al., 2011), the equilibrated system was duplicated along the z direction and transmembrane potential gradients were generated by introducing a charge difference of 2 K^+ ions between the two compartments separated by the two lipid bilayers. During the MD simulations, the number of the ions was kept constant by an additional algorithm (Kutzner et al., 2011). The resulting membrane potential can be calculated by double-integration of the charge distribution using the Poisson equation as implemented in the GROMACS tool g_potential (Tieleman and Berendsen, 1996). In the production simulations, two ionic configurations were employed in the starting structures: KWKKK and KKKKK (S0, S1, S2, S3, S4), respectively. During the simulations a permeation event was counted when an ion moved from the cavity to the filter, and another ion left the S0 position.

References

Hess, B., Kutzner, C., van der Spoel, D., and Lindahl, E. (2008). GROMACS 4: algorithms for highly efficient, load-balanced, and scalable molecular simulation. J Chem Theory Comput *4*, 435–447.

Hornak, V., Abel, R., Okur, A., Strockbine, B., Roitberg, A., and Simmerling, C. (2006). Comparison of multiple Amber force fields and development of improved protein backbone parameters. Proteins *65*, 712-725.

Joung, I.S., and Cheatham, T.E., 3rd. (2008). Determination of alkali and halide monovalent ion parameters for use in explicitly solvated biomolecular simulations. J Phys Chem B *112*, 9020–9041.

Berger, O., Edholm, O., and Jähnig, F. (1997). Molecular dynamics simulations of a fluid bilayer of dipalmitoylphosphatidylcholine at full hydration, constant pressure, and constant temperature. Biophys J *72*, 2002–2013.

Darden, T., York, D., and Pedersen, L. (1993). Particle mesh Eward: An N log(N) method for Ewald sums in large systems. J Chem Phys *98*, 10089.

Essmann, U., Perera, L., Berkowitz, M. L., Darden, T., Lee, H., and Pedersen, L. G. (1995). A smooth particle mesh Ewald method. J Chem Phys *103*, 8577-8593.

Bussi, G., Donadio, D., and Parrinello, M. (2007) Canonical sampling through velocity rescaling. J Chem Phys *126*, 014101.

Berendsen, H. J. C., Postma, J. P. M., van Gunsteren, W. F., Dinola, A., and Haak, J. R. (1984). Molecular dynamics with coupling to an external bath. J Chem Phys *81*, 3684-3690.

Hess, B., Bekker, H., Berendsen, H. J. C., and Fraaije, J. G. E. M. (1997). LINCS: A linear constraint solver for molecular simulations. J Comput Chem *18*, 1463-1472.

Feenstra, K. A., Hess, B., and Berendsen, H. J. C. (1999). Improving efficiency of large time-scale molecular dynamics simulations of hydrogen-rich systems. J Comput Chem *20*, 786-788.

Brohawn, S. G., Campbell, E. B., and MacKinnon, R. (2013). Domain-swapped chain connectivity and gated membrane access in a Fab-mediated crystal of the human TRAAK K+ channel. Proc Natl Acad Sci USA *110(6)*, 2129-2134.

Sato, C. S., Fasnacht, M., Zhu, J., Forrest, L., and Honig, B. (2008). Loop modeling: Sampling, filtering, and scoring. Proteins *70(3)*, 834-843.

Wolf, M.G., Hoefling, M., Aponte-Santamaría, C., Grubmüller, H., and Groenhof, G. (2010). g_membed: Efficient insertion of a membrane protein into an equilibrated lipid bilayer with minimal perturbation. J Comput Chem *31,* 2169–2174.

Kutzner, C., Grubmüller, H., de Groot, B.L., and Zachariae, U. (2011). Computational electrophysiology: the molecular dynamics of ion channel permeation and selectivity in atomistic detail. Biophys J *101*, 809–817.

Tieleman, D., and Berendsen, H. J. C. (1996). Molecular dynamics simulations of a fully hydrated dipalmitoylphosphatidylcholine bilayer with different macroscopic boundary conditions and parameters. J Chem Phys *105*, 4871–4880.
